# Brain Tumor Segmentation Using an Ensemble of 3D U-Nets and Overall Survival Prediction Using Radiomic Features

**DOI:** 10.3389/fncom.2020.00025

**Published:** 2020-04-08

**Authors:** Xue Feng, Nicholas J. Tustison, Sohil H. Patel, Craig H. Meyer

**Affiliations:** ^1^Department of Biomedical Engineering, University of Virginia, Charlottesville, VA, United States; ^2^Department of Radiology and Medical Imaging, University of Virginia, Charlottesville, VA, United States

**Keywords:** brain tumor segmentation, ensemble, 3D U-net, deep learning, survival prediction, linear regression

## Abstract

Accurate segmentation of different sub-regions of gliomas such as peritumoral edema, necrotic core, enhancing, and non-enhancing tumor core from multimodal MRI scans has important clinical relevance in diagnosis, prognosis and treatment of brain tumors. However, due to the highly heterogeneous appearance and shape of these tumors, segmentation of the sub-regions is challenging. Recent developments using deep learning models has proved its effectiveness in various semantic and medical image segmentation tasks, many of which are based on the U-Net network structure with symmetric encoding and decoding paths for end-to-end segmentation due to its high efficiency and good performance. In brain tumor segmentation, the 3D nature of multimodal MRI poses challenges such as memory and computation limitations and class imbalance when directly adopting the U-Net structure. In this study we aim to develop a deep learning model using a 3D U-Net with adaptations in the training and testing strategies, network structures, and model parameters for brain tumor segmentation. Furthermore, instead of picking one best model, an ensemble of multiple models trained with different hyper-parameters are used to reduce random errors from each model and yield improved performance. Preliminary results demonstrate the effectiveness of this method and achieved the 9th place in the very competitive 2018 Multimodal Brain Tumor Segmentation (BraTS) challenge. In addition, to emphasize the clinical value of the developed segmentation method, a linear model based on the radiomics features extracted from segmentation and other clinical features are developed to predict patient overall survival. Evaluation of these innovations shows high prediction accuracy in both low-grade glioma and glioblastoma patients, which achieved the 1st place in the 2018 BraTS challenge.

## Introduction

Gliomas are the most common primary brain malignancies, with different degrees of aggressiveness, variable prognosis and various heterogeneous histological sub-regions, i.e., peritumoral edema, necrotic core, enhancing, and non-enhancing tumor core (Wrensch et al., 2002; Louis et al., 2016). This intrinsic heterogeneity of gliomas is also portrayed in their radiographic phenotypes, as their sub-regions are depicted by different intensity profiles disseminated across multimodal MRI (mMRI) scans, reflecting differences in tumor biology (Cha, 2006; Upadhyay and Waldman, 2011). Quantitative analysis of imaging features such as volumetric measures after manual/semi-automatic segmentation of the tumor region has shown advantages in image-based tumor phenotyping over traditionally used clinical measures such as largest anterior-posterior, transverse, and inferior-superior tumor dimensions on a subjectively-chosen slice (Kumar et al., 2012; Gillies et al., 2016). Such phenotyping may enable assessment of reflected biological processes and assist in surgical and treatment planning. For brain tumors, including sub-regions, segmentation is challenging due to their highly heterogeneous appearance and shape, which may be further complicated by imaging artifacts such as motion and/or field inhomogeneity.

In recent years, deep convolutional neural networks (DCNN) have demonstrated effectiveness in natural and medical image segmentation tasks, including those associated with brain tumor segmentation (Akkus et al., 2017; Havaei et al., 2017; Iqbal et al., 2018; Naceur et al., 2018). However, one main issue in DCNN methods is the reliance on a large number of training data with expert annotations, which are often difficult to obtain, especially from multiple institutions. To provide such a dataset to the scientific community and a platform to compare and evaluate different automatic segmentation algorithms for brain tumors, the Multimodal Brain Tumor Segmentation Challenge (BraTS) was organized using multi-institutional pre-operative MRI scans for the segmentation of intrinsically heterogeneous brain tumor sub-regions (Menze et al., 2015; Bakas et al., 2017a,b,b), with the dataset growing every year. In the 2018 challenge, 285 training cases, 66 validation cases, and 191 testing cases were provided. Not surprisingly, DCNN-based models have quickly become the mainstream in BraTS challenges (Bakas et al., 2018). Similar to classification networks, one common DCNN method for segmentation is to use the extracted small patches to predict the class for the center voxel and slide these patches to cover the entire volume; to improve the classification accuracy of the center voxel, multi-scale patches with different receptive field sizes can be extracted simultaneously as in Kamnisas et al. (2017). In contrast, U-Net is a widely used network structure that consists of a contracting path to capture context and a symmetric expanding path that enables precise localization and segmentation for the entire input image (Ronneberger et al., 2015). If the input images and the corresponding output label maps are 3-dimensional (3D), the original U-Net construction can be extended by replacing 2D operations with their 3D counterparts (Cicek et al., 2016). However, in such cases the requirement for memory and computation speed is greatly increased so that it may not be possible to use the entire 3D volume as the input and output. To address this issue, one method is to extract smaller 3D patches as the network input and generate the label maps corresponding to these patches (Li et al., 2018). To achieve a good segmentation performance, data augmentation and optimization of patch extraction strategy and network hyper-parameters are often performed. However, in practice, it is very challenging to achieve a single “optimized” model and it is possible that any model can suffer from random errors. Using a similar concept as in traditional machine learning tasks, an ensemble of multiple models can generally improve the classification/segmentation accuracy as individual models may make different errors and by averaging or majority voting, the final number of errors can be reduced (Tan and Gilber, 2003). In this study we propose the use of an ensemble of 3D U-Nets with different hyper-parameters for brain tumor segmentation. For each 3D U-Net, the smaller 3D patches will be extracted to minimize memory overhead. To avoid extracting too many background patches and not learning sufficient information to segment tumors, a customized probability function is used to guide the patch extraction process. Furthermore, during testing, a sliding window approach is used to predict class labels with overlap between patches as a testing augmentation method to improve accuracy. On the network structure, although many new methods have been proposed that show superior performance than the U-Net in segmentation tasks, such as the densely connected network (Dense-Net) (Jegou et al., 2016; Stawiaski, 2019), a recent paper claimed that optimization on various training and testing details based on vanilla U-Net can yield robust and superior performance (Isensee et al., 2018). In our study we will compare the U-Net with Dense-Net for this task when other strategies are kept the same.

Survival prediction has a very high clinical value in prognosis and patient management. In the BraTS challenge, to demonstrate one potential clinical application of the segmentation results, the task to predict patient overall survival measured in days was also included. Additional data including patient age and resection status was provided. For training cases, the overall survival was also available for part of the dataset. Although complicated models such as DCNN or random forests (Tustison et al., 2015) can be used to capture sophisticated relationships between the input features and the output of overall survival, one main issue with these methods is overfitting, especially in this task as the training data is very small compared with the huge number of possible input features. Furthermore, the radiomics features are often difficult to explain as they lack direct clinical correspondence. Using the segmentation method proposed in this study, the sub-regions of brain tumor are expected to be accurately segmented so that various quantitative features can be calculated. To reduce overfitting, we will utilize the quantitative results and a robust linear model while limiting the number of extracted features. The correlations of these features with overall survival will also be analyzed.

## Methods

For the brain tumor segmentation task, the steps in our proposed method include pre-processing of the images, patch extraction, training multiple models using a generic 3D U-Net structure with different hyper-parameters, deployment of each model for the full volume prediction and the final ensemble step. For the survival prediction task, the steps include feature extraction, model fitting, and deployment. Data description and methodological details are provided in the following sections.

### Dataset and Image Pre-processing

The datasets used in this study are provided by the BraTS challenge organizers and contains multiple-institutional clinically-acquired pre-operative multimodal MRI scans of glioblastoma (GBM/HGG) and low-grade glioma (LGG) containing (a) native (T1) and (b) post-contrast T1-weighted (T1Gd), (c) T2-weighted (T2), and (d) Fluid Attenuated Inversion Recovery (FLAIR) volumes. They were acquired with different clinical protocols and various scanners. All the imaging datasets have been segmented manually, by one to four raters, following the same annotation protocol, and their annotations were approved by experienced neuro-radiologists. Annotations comprise the GD-enhancing tumor (ET—label 4), the peritumoral edema (ED—label 2), and the necrotic and non-enhancing tumor core (NCR/NET—label 1). During training, 285 imaging cases with annotations were provided to all challenge participants. An additional 66 cases were used as validation data which did not include ground truth labels. Additionally, participants were able to upload their predictions multiple times and get the corresponding evaluation results. During the testing phase, 191 cases were provided and the teams could only upload their results once in a 48-h period and receive the final score.

To accommodate for the differences in imaging protocols, pre-processing was performed by the challenge organizers. The images from different MR sequences of the same subject were first co-registered to the same anatomical template, the SRI24 multichannel atlas of normal adult human brain (Rohlfing et al., 2010), followed by interpolation and zero-padding to the same resolution (1 mm^3^) and same matrix size (240x240x155). The field-of-view (FOV) was then unified accordingly (240 mm along the left-right and anterior-posterior directions and 155 mm along the superior-inferior direction). Brain extraction was also performed using the method described in Bauer et al. (2012). To improve the homogeneity and suppress noise, N4 bias-correction (Tustison et al., 2010) and denoising using non-local means (Manjon et al., 2010) are often used in various studies. However, although these pre-processing steps can yield visually improved image quality, as shown in our previous study (Feng et al., 2018), we did not achieve an improved segmentation result on the validation data set. Considering the bias-correction and denoising algorithms are computationally intensive and time-consuming, we did not perform these two steps. To unify the intensity range, each MR sequence is scaled to be between 0 and 1.

To achieve the second task to predict patient overall survival, during training, 163 cases out of the total 285 had age, resection status and survival information available. However, the cases from The Cancer Imaging Archive (TCIA) and a few other cases did not have the resection status available so they were labeled as “NA.” For all other cases, the status was either Gross Total Resection (GTR) or Subtotal Resection (STR). The survival time was given in days. During validation, 53 cases with age and resection status were provided. Similar with the segmentation task, the participants could upload the prediction multiple times. However, only 28 cases with resection status GTR were evaluated. During testing, 130 cases were provided and 77 were evaluated.

### Non-uniform Patch Extraction

For simplicity, we will use foreground to denote all tumor pixels and background to denote the rest. There are several challenges in directly using the whole image as the input to a 3D U-Net: (1) the memory of a moderate GPU is often 12 Gb so that in order to fit the model into the GPU, the network needs to greatly reduce the number of features and/or the layers, which often leads to a significant drop in performance as the expressiveness of the network is much reduced; (2) the training time will be greatly prolonged since more voxels contribute to calculation of the gradients at each step and the number of steps cannot be proportionally reduced during optimization; (3) as the background voxels dominate the whole image, the class imbalance will cause the model to focus on background if trained with uniform loss, or prone to false positives if trained with weighted loss that favors the foreground voxels. Therefore, to more effectively utilize the training data, smaller patches were extracted from each subject. As the foreground labels contain much more variability and are the main targets to segment, more patches from the foreground voxels should be extracted.

In implementation, during each epoch of the training process, a random patch was extracted from each subject using non-uniform probabilities. In extraction, the voxel was first chosen as the center of the patch and the corresponding patch was extracted based on the desired size. To make sure that each extracted patch is within the whole image so that no padding is required, the voxels close to the edge of the image were excluded when determining the patch center. From all voxels valid to be the patch center, the sampling was performed based on the probability function *p*_*i,j,k*_ calculated using the following equation:

(1) $$\begin{array}{r} p_{i,j,k}=\frac{s_{i,j,k}}{\sum_{i,j,k}s_{i,j,k}} \end{array}$$

in which *s*_*i,j,k*_ = 1 for all voxels with maximal intensity lower than the 1st percentile, *s*_*i,j,k*_ = 6 for all foreground voxels and *s*_*i,j,k*_ = 3 for the rest. These values were picked to greatly favor the tumor regions and slightly favor the regions with normal brain tissue compared with the background voxels. However, the exact ratio was determined empirically without rigorous tuning. For each training iteration, one patch was extracted using this method. Since normal brain images are symmetric along the left-right direction, a random flip along this direction was made after patch extraction. No other augmentation was applied.

Before training, the per-input-channel mean and standard deviation of extracted patches were calculated by running the extraction process 400 times, with each time using a randomly selected training subject. The extracted patches were then normalized by subtracting the mean and dividing by the standard deviation along each input channel.

### Network Structure and Training

**Figure 1:**
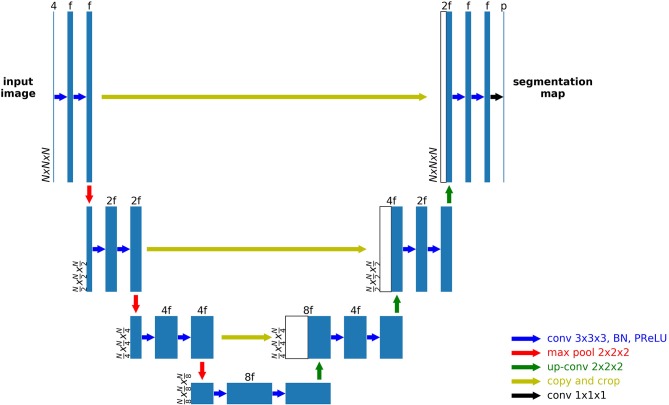
An example 3D U-Net structure with 3 encoding and 3 decoding blocks.

A 3D U-Net based network was used as the general structure, as shown in Figure 1. Zero padding was used to make sure the spatial dimension of the output is the same with the input. For each encoding block, a VGG like network (Simonyan and Zisserman, 2014) with two consecutive 3D convolutional layers of kernel size 3 followed by the activation function and batch norm layers were used. The parametric rectilinear function (PReLU) (Xu et al., 2015), given as:

(2) $$\begin{array}{r} f\left(x\right)=\max\left(0,x\right)-\alpha \;\max\left(0,-x\right) \end{array}$$

was used as the activation function (with trainable parameter α). The number of features was doubled while the spatial dimension was halved with every encoding block, as in the conventional U-Net structure. A dropout layer with ratio 0.5 was added after the last encoding block. Symmetric decoding blocks were used with skip-connections from corresponding encoding blocks. Features were concatenated to the de-convolution outputs. The extracted segmentation map of the input patch was expanded to the multi-class the ground truth labels (3 foreground classes and the background). Cross entropy was used as the loss function. In addition to a uniform loss among all classification labels, the weighted loss, in which different labels can be assigned with different weights, was also used.

It is shown that a wider network with large number of features and a deeper network can increase the expressiveness and thus performance of the network (Wu et al., 2016); furthermore, the larger the patch size, the more spatial information to be used in one patch; however, as mentioned before, the memory of the GPU is often a limiting factor with 3D inputs. In our study, we balanced the three parameters (number of encoding/decoding blocks, input features at the first layer and patch size) to make sure that the GPU memory is sufficient while favoring one in one model. Specifically, if the patch size is increased, to keep the same rule of doubling the number of filters every block, the number of blocks cannot be more than 3 without exceeding GPU memory. The exact choice of these parameters was made empirically with the general principle to be as different as possible to reduce the correlations of random errors by a single parameter set. In addition, the weighted loss function, which favors the foreground voxels, can often improve the sensitivity but sacrifice specificity as it punishes more for missed foreground segmentations. Therefore, for each combination of these parameters, we used both the weighted and uniform loss functions. Although the increase of the number of models may further benefit the final results, in a way that is similar with more averages, the time for training and testing will also increase proportionally. Therefore, a total of six model was selected, with detailed parameters shown in Table 1. N denotes the input patch size, M denotes the number of encoding/decoding blocks and f denotes the input features at the first layer. For the weighted loss function, 1.0 was used for background and 2.0 was used for each of the foreground classes.

**Table 1:** Detailed parameters for all 6 3D U-Net models.

**Model#**	**M**	**N**	**f**	**Loss Type**
1	3	64	96	Uniform
2	3	64	96	Weighted
3	4	64	96	Uniform
4	4	64	96	Weighted
5	3	80	64	Uniform
6	3	80	64	Weighted

Training was performed on a Nvidia Titan Xp GPU with 12 Gb memory. Six hundred forty epochs were used. As mentioned earlier, during each epoch, only one patch was extracted from every subject. Subject orders were randomly permuted every epoch. Implementation was based on the TensorFlow framework. Batch size was set to 1 during training. During testing, due to the sensitivity associated with smaller batch sizes, all batch norm layers did not use the running statistics but the statistics of the batch itself. This is the same as instance normalization (Ulyanov et al., 2016) when the batch size is 1 as it normalizes each feature map with its own mean and standard deviation. The Adam optimizer was used with an initial learning rate of 0.0005 without further adjustments during training as it can self-adjust the rate of gradient update so that no manual reduction of learning rate is necessary (Kingma and Ba, 2014). The total training time was about 60 h.

### Deployment of Each Segmentation Model and Ensemble

Although the fully convolutional segmentation network can be applied to the input images of any size, due to the fact that the whole network with the entire image as the input cannot fit into the memory during deployment, a sliding window approach needs to be used to get the output for each subject. However, as significant padding was made to generate the output label map at the same size as the input, boundary voxels of a patch were expected to yield unstable predictions when sliding the window across the whole image without overlaps. To alleviate this problem, a stride size at a fraction of the window size was used and the output probability was averaged. In implementation, the deployment window size was chosen to be the same as the training window size, and the stride was chosen as ½ of the window size. For each window, the original image and left-right flipped image were both predicted, and the average probability after flipping back the output of the flipped input was used as the output. Therefore, each voxel, except for a few on the edge, will be predicted 16 times when sliding across all directions. Although smaller stride sizes can be used to further improve the accuracy with more averages, the deployment time will be increased 8 times for every ½ reduction of the window size and thus quickly becomes unmanageable. Using the parameters as mentioned on the same GPU, it took about 1 min to generate the output for the entire volume per subject. Instead of performing a thresholding on the probability output to get the final labels, the direct probability output after the last convolutional layer was saved for each model as a measure of “confidence” for each model.

The ensemble modeling process was rather straightforward. The probability output of all classes from each model was averaged to get the final probability output. The class with the highest probability was selected as the final segmentation label for each voxel.

### Comparison of U-Net and Dense-Net

The Dense-Net was implemented following the standard structure as in Jegou et al. (2016). Specifically, the block number was 4, layers per block was 12 and the growth rate was 12. In terms of architecture, the Dense-Net-BC (further compression) was used. The uniform cross entropy function was used as the loss function. As a fair comparison, only the U-Net with 4 encoding/decoding blocks and uniform loss function (model 3 in Table 1) was compared. The patch extraction and augmentation were kept the same for the two models. As the evaluation using the BraTS validation and testing datasets requires submission to the server of the BraTS organizers, which has a limit on the number of allowed submissions, we only used the BraTS training dataset and randomly split it with a 3:2 ratio for training and validation in this comparison experiment.

### Survival Prediction

To predict the post-surgery survival time measured in days, extracted imaging features and non-imaging features were used to construct a linear regression model. As MR images often exhibit variations in imaging intensity and contrast, the intensity values of the images were not directly used in our survival modeling. Instead, six simple volumetric features were calculated from the segmented labels of the three tumor sub-regions: the enhancing tumor core, non-enhancing and necrotic region and edema, with two features per region. During training, the ground truth label maps were used; during validation and testing, the automatically segmented label maps were used. For each foreground class, the volume (V) was determined by summing up the voxels whereas the surface area (S) was calculated by summing up the magnitude of the gradients along three directions, as described in the following equations

(3) $$\begin{array}{r} V_{ROI}=\underset{i,j,k}{\sum }s_{i,j,k} \end{array}$$

(4) $$\begin{array}{r} S_{ROI}=\sum_{i,j,k}s_{i,j,k}\sqrt{{\left(\frac{\partial s}{\partial i}\right)}^{2}+{\left(\frac{\partial s}{\partial j}\right)}^{2}+\;{\left(\frac{\partial s}{\partial k}\right)}^{2}} \end{array}$$

in which ROI denotes a specific foreground class and *s*_*i,j,k*_ = 1 for voxels that are classified to belong to this ROI and *s*_*i,j,k*_ = 0 otherwise. The volume represents the size of each sub-region and thus may reflect the severity of the tumor. It is expected that the larger the volume, the poorer the prognosis. The surface area is another measure of the size; however, together with volume, it can also serve as a measure for the shape. Given a fixed volume, the more irregular the shape, the larger the surface area; therefore, a larger surface area may indicate the aggressiveness of the tumor and the increased difficulty in surgery.

Age and resection status were used as non-imaging clinical features. As there were two classes of resection status and many missing values of this status, a two-dimensional feature vector was used to represent the status, given as GTR: (1, 0), STR: (0, 1), and NA: (0, 0). A linear regression model was employed after normalizing each input feature to zero mean and unit standard deviation. As the input feature size is 9, the risk for overfitting is greatly reduced.

For evaluation, in addition to mean and median square error of survival time predictions, the classification of subjects as long-survivors (e.g., >15 months), short-survivors (e.g., <10 months), and mid-survivors (e.g., between 10 and 15 months) was performed. For the challenge, ranking of the participating teams was based on accuracy (i.e., the number of correctly classified patients) with respect to this grouping.

## Results

### Comparison of U-Net and Dense-Net

**Figure 2:**
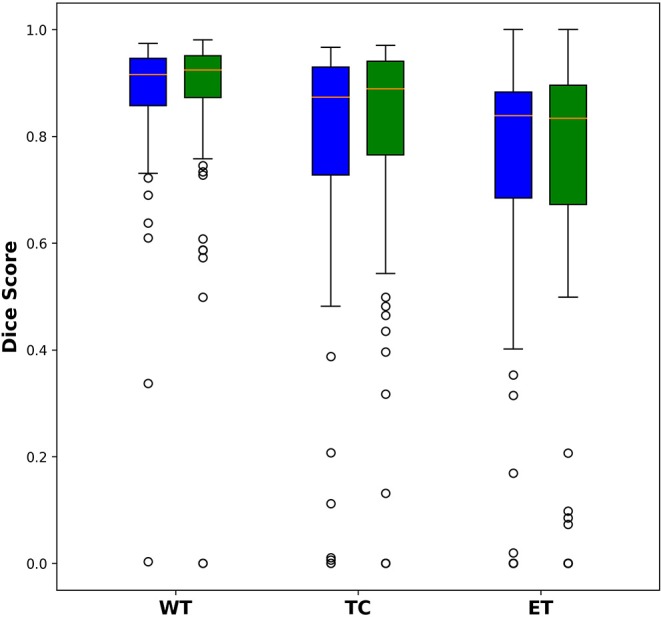
Comparison of dice indexes using U-Net and Dense-Net. Green bars show the results using U-Net; blue bars show the results using Dense-Net. The two models have very similar performances without any statistically significant differences.

Among the 285 training subjects, 171 were used for training the two models and 114 were used for testing. The dice indexes of the enhanced tumor (ET), whole tumor (WT) and tumor core (TC) were calculated and compared, as shown in Figure 2. The blue bars show the results from U-Net and the green bars show those from Dense-Net. The two methods yield very similar performances with the Dense-Net having slightly better performance in tumor core. However, the paired Student's *t*-test was performed between the two methods and showed no statistically significant differences when the threshold of *p*-value was set at 0.05.

### Brain Tumor Segmentation

All 285 training subjects were used in the training process. 66 subjects were provided as validation. The dice indexes, sensitivities and specificities, 95% Hausdorff distances of ET, WT, and TC were automatically calculated after submitting to the CBICA's Image Processing Portal. ET corresponds to label 4 in the direct output label maps; WT is the union of all non-background label maps including label 1, 2, and 4; TC is the union of ET and NCR/NET, or label 1 and 4. With multiple submissions, we were able to compare the performances of each individual model and the final ensemble.

Table 2 shows the mean Dice scores (Dice) and 95% Hausdorff distances (Dist) of ET, WT and TC in mm for the 6 individual models and the ensemble of them. The model with the best performance of each metric is highlighted. For the evaluation, sensitivity and specificity were also calculated to determine over- or under-segmentations of tumor sub-regions. Detailed descriptions of the evaluation metrics were provided in Menze et al. (2015). As we found that sensitivity and specificity were highly correlated with the Dice indexes, they are not included in the table. The best performance of each evaluation metric is highlighted. For WT, all 3D U-Net models perform similarly, except for a slightly worse performance with model 4. However, model 4 has the highest Dice for ET. The rankings based on Dice scores are also not consistent with the rankings based on the distance measures. This shows that no single parameter set has clear advantage over others. However, the ensemble of them has the best overall Dice scores as compared with each individual model. Paired student's *t*-tests were performed between each model and the ensemble on Dice scores with the red scores showing statistically inferior performances of one model compared with the ensemble (*p* < 0.05). For WT, model 1–5 all showed significant inferior performances. For model 6, although no statistical significance was found, the *p*-values were close to 0.05. The distance metrics show a wider range and the ensemble does not achieve the smallest values. However, as the Haudorff distance is largely determined by the “worst” pixels, it may be less reliable in obtaining an overall performance evaluation as compared with Dice scores. Despite this, the metrics in the ensemble method for all three sub-regions are all on the lower end, showing increased robustness. It is also noticed that weighted cross-entropy loss has high sensitivity but lower specificity compared with the uniform counterpart, which is likely due to the fact that by assigning more weights to the foreground, the network tends to be more aggressive in assigning foreground labels.

**Table 2:** Performances of each individual model and the ensemble.

**Model #**	**Dice_ET**	**Dice_WT**	**Dice_TC**	**Dist_ET**	**Dist_WT**	**Dist_TC**
1	0.7839	0.9061	0.8233	4.0496	4.0401	6.5389
2	0.7681	0.9070	0.8126	4.2215	6.1359	**6.0561**
3	0.7538	0.9072	0.8236	4.7615	5.7021	9.0000
4	0.7874	0.9001	0.8088	**3.9195**	6.3093	6.9586
5	0.7704	0.9061	0.8227	4.0314	4.7068	6.5905
6	0.7819	0.9097	0.8217	3.9368	**3.6666**	6.3705
Ensemble	**0.7946**	**0.9114**	**0.8304**	3.9679	3.7842	6.5234

*Bold values show the model with the best performance of each metric*.

**Figure 3:**
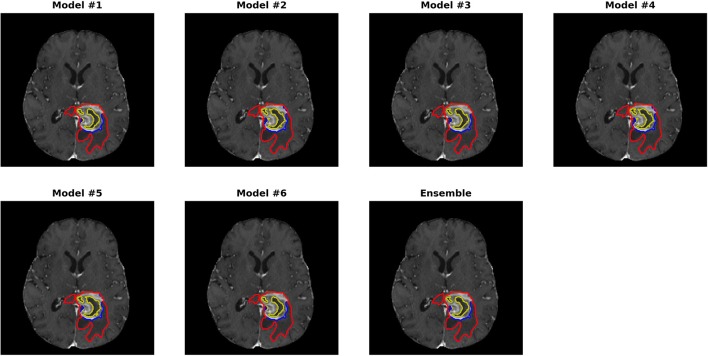
Automatically segmented sub-regions from models 1–6 and the ensemble model. The underlying image is the corresponding T1Gd from the validation case “Brats18_CBICA_BHF_1.” Red, yellow and blue delineate the predicted boundaries of the total tumor, enhanced tumor core, and peritumoral edema, respectively.

**Figure 4:**
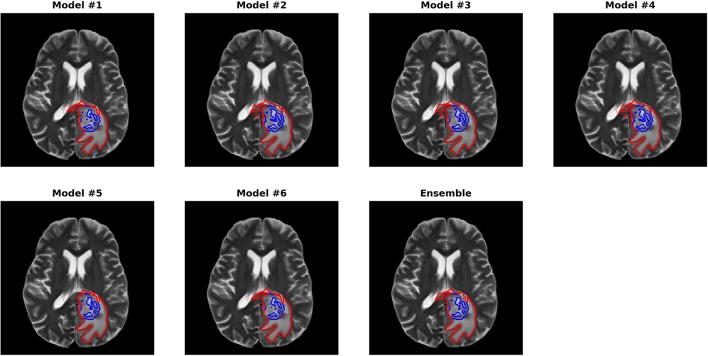
Automatically segmented sub-regions from model 1–6 and the ensembled model. The underlying image is the corresponding T2 from the validation case “Brats18_CBICA_BHF_1.” Red, yellow and blue delineate the predicted boundaries of the total tumor, enhanced tumor core, and peritumoral edema, respectively.

Figures 3, 4 show two slices (axial slice 76 and 81) of the automatically segmented labels overlaid on the T1Gd and T2 images, respectively. The showed case was “Brats18_CBICA_BHF_1” and was randomly selected from the validation dataset for demonstration. A single model may suffer from under- or over-segmentation while the average of multiple models achieves a more stable performance, which is also closer to the ground-truth, as shown with the improved Dice scores. Furthermore, the ensemble of all 6 models yields a much smoother boundary for different sub-regions and eliminates a few isolated regions, which are likely false positives.

In 191 testing cases, as only one submission was allowed, we submitted the final ensemble results for evaluation. The mean Dice scores for ET, WT and TC were 0.754, 0.878, and 0.799 and the 95% Hausdorff distances were 20.29, 7.41, and 22.06 mm, respectively. It is also noted that 2 of the testing cases failed to predict any tumor voxels, resulting in Dice scores of 0. Compared with validation cases, the average performance for testing cases was much worse.

The paper published by the challenge organizers (Bakas et al., 2018) summarized the performance by all 63 participating teams, including ours. The ranking was based on the testing cases as only one submission is allowed to avoid learning from the submissions. Our team (xfeng) achieved the 9th place in the segmentation task [Figure 7 in Bakas et al. (2018)]. However, the differences among the top teams were relatively small.

### Overall Survival Prediction

**Figure 5:**
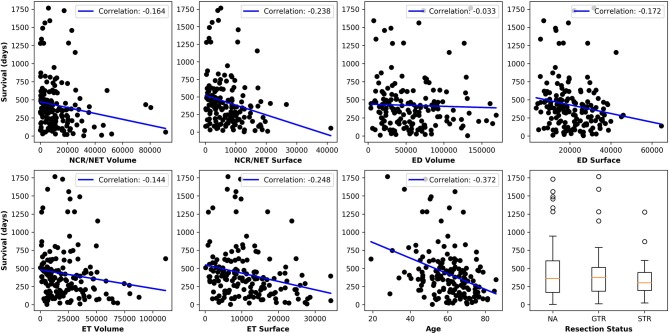
Relationships between each extracted feature and the overall survival. The correlation coefficients are shown as well. Age shows the strongest negative correlation with survival and all imaging features show moderate to very weak negative correlations.

**Figure 6:**
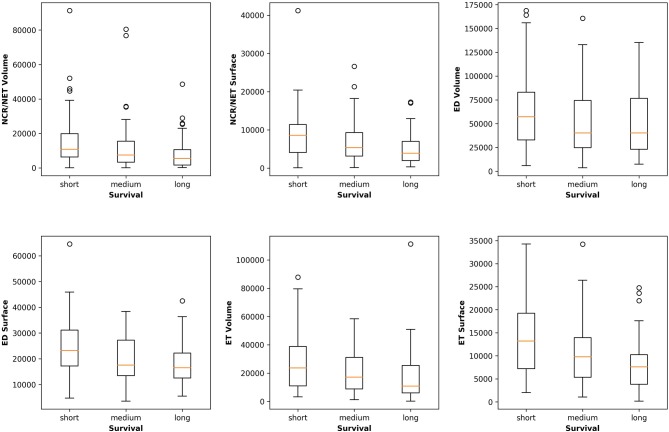
Relationships between the volume and surface features and the binned overall survival (short-, medium-, and long-term survival). The general trend shows that the smaller the volume and surface, the longer the survival.

Figure 5 plots the extracted features against the overall survival in the training data. The correlation coefficients between the six radiomic features from images and the overall survival were also calculated as well as between age and the survival. Negative correlations between imaging features and the survival are observed, indicating that the larger the specific tumor sub-regions, the shorter the survival will be. To better illustrate this trend, we binned the overall survival into the short-term (<10 months), medium-term (10–15 months) and long-term (>15 months) and drawn the box plots for survivals, as shown in Figure 6. The general trend is consistent with the previous results, showing that the larger the volume and surface, the worse the prognosis. The correlation between age and survival is also expected. Furthermore, the correlation between age and survival is the strongest among all selected features. For resection status, patients who underwent GTR have longer survival rates than the STR patients. However, no statistical differences were found using a Student's *t*-test.

A multivariate linear regression model was trained with all the features from 163 training subjects. For the 28 validation cases, the accuracy was 0.321. The mean and median errors were 314.8 and 278.85 days, respectively. For the 77 testing cases, accuracy was 0.61 corresponding to mean and median errors of 481.4 and 185.22 days, respectively. It should be noted that the accuracy for the testing cases was much higher than for the validation cases. We did not use the validation cases to tune any parameters in training the model due to potential overfitting. Our testing performance ranked 1st among all participants, indicating the robustness of the linear model. Compared with other participating teams, who used radiomics and/or machine learning based modeling, this simple strategy yielded the best accuracy. It is noted that one team used the age as the only predictor and used a linear regression model similar to our method and achieved the 3rd place in survival task, as summarized in Bakas et al. (2018).

## Discussion and Conclusions

In this paper we developed a brain tumor segmentation method using an ensemble of 3D U-Nets. Six networks with different numbers of encoding/decoding blocks, input patch sizes and different weights for loss were trained and ensembled together by averaging the final prediction probabilities. The results showed improvements with the ensemble model compared with any of the single models. For the survival prediction task, we extracted six simple features from the segmentation labels and used a multivariate linear regression by combining them with non-imaging clinical features such as age and resection status. The survival prediction achieved 1st place among all challenge participants.

In terms of network structure, we found it very difficult to pick the “best” model and/or hyper-parameter set since most models perform very similarly. The comparison between U-Net and Dense-Net showed that it is hard to pick a clear winner for network structure. It is indeed one disadvantage of DCNN as the “black-box” nature of the network makes it challenging to analyze the effect of network structure and parameter except from the final performance. Furthermore, the extremely long computation times and randomness in training the model and selected validation datasets makes comparison of different models difficult. In this paper, we empirically determined a few design options such as the usage of 3D U-Net and non-uniform patch extraction. Multiple models with architectural variation can form an ensemble to overcome random errors made by any individual model. Similar to using averages in measurements to improve signal-to-noise ratio, in which the marginal increase of performance can reduce as the number of averages increases, we aim to strike a balance between training and validation time and the expected performance. The ensemble yielded an improved performance in both quantitative measures and visual examination; however, one limitation of our approach is the lack of objective measures to achieve optimal combination of models. Instead, we empirically determined number of models to be 6 and chose the corresponding hyperparameters. An interesting alternative is to use grid search to gain an optimal set of hyperparameters, which is currently a popular research topic; however, one possible concern is that this may lead to overfitting as the validation set is much smaller (66 cases) compared with the training and testing dataset; to mitigate this concern, N-fold cross validation can be used in combination with the grid search method, which will be performed in future studies.

Compared with the patch-based model that only predicts the center pixel, the 3D U-Net predicts the segmentation label map for the full input. As it is limited by the GPU memory to use the full image as the input, smaller patches are extracted. However, this can lead to reduced receptive field, which is even worse for the pixels on the edge as only half of the receptive field contains information. We hypothesize that with a much larger patch size such as 128x128x128, the performance can be improved, however, the majority of GPUs only have 12 Gb memory, which cannot deal with such an input without significantly sacrificing the network complexity. To overcome the reduced receptive field of the edge pixels, we used significant overlap during deployment in a sliding windows fashion and average the output, which shows performance improvement.

For pre-processing, although in many studies the bias correction was commonly used, as in our previous experiment, we did not find any significant benefit in the proposed method. Although bias correction can greatly improve the quality of the image by removing inhomogeneity artifacts and thus segmentation performance for any intensity-based method, DCNN may be able to learn and overcome any bias in the image so that it may not be necessary to pre-compensate for it. As it is time-consuming to run the bias correction, we did not perform this step in our final experiments. However, additional experiments on other datasets are required to continue the investigation on this topic.

**Figure 7:**
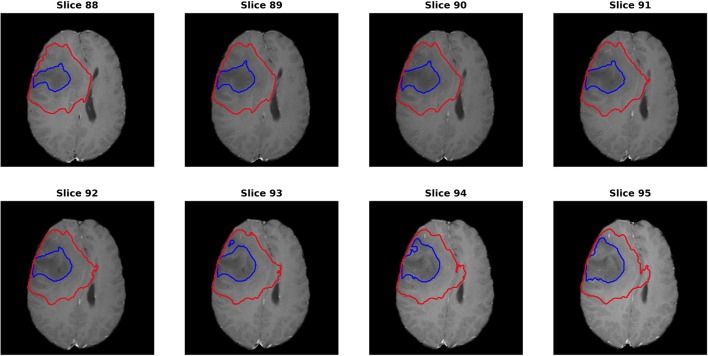
One case (“Brats18_TCIA10_195_1”) from the validation dataset showing failed segmentation for ET region on post-contrast T1. Red and blue show the boundaries for whole tumor and edema, respectively. No enhanced region is detected. It is indeed very difficult to determine the exact enhanced areas in the images, even for human experts.

For the segmentation results, as we can get the evaluation for each individual case, it is noted that the median metrics were significantly higher than the mean metrics. For example, the median Dice scores were 0.870, 0.926, and 0.911 for ET, WT, and TC in the final ensemble model. It makes sense in that the theoretical maximum Dice score is 1 and minimum Dice score is 0. However, we noted that in several cases, the Dice scores were as low as 0 for ET and TC, meaning that the model completely missed the corresponding regions. Figure 7 shows an example (“Brats18_TCIA10_195_1”) with 0 Dice score for ET with post-contrast T1. Red and blue show the contours for WT and edema, respectively. No ET is detected in this case. However, it is indeed very difficult to identify the enhanced regions as the contrast enhancement is weak. Most of the subjects had the WT Dice score larger than 0.9, indicating very high segmentation quality. However, one case had a much lower Dice of 0.63. A careful examination showed that this case predicted a very small tumor region and the contrast was visually weak. It shows that although in most cases the automatic segmentation yields very accurate result, in difficult cases with reduced contrast and/or small tumor region, the automatic result may be sub-optimal and manual expert examination and correction is still required.

Comparing the testing with validation cases, we noticed a significant gap in performance. Due to the design of the challenge, the participants can submit multiple times for the validation cases to gain any performance improvements so that the model may overfit on the validation cases; however, in our study we did not use the validation cases to perform any hyper-parameter tuning to select an optimal model. Therefore, the performance differences are likely due to more difficult cases in the testing dataset, including the two that the model completely failed. One possible reason is that the testing data covers a wide range of MR imaging protocols and field strength, some even with moderate to severe artifacts due to motion and/or inhomogeneity in one or multiple sequences, causing difficulty in achieving a consistent segmentation performance. Further investigation to continue to improve the performance and robustness of the model, especially for these difficulty cases, will be performed.

Our segmentation method ranked 9th in the challenge. The 1st place winner used a patch size of 128x128x128 with autoencoder regularization (Myronenko, 2019) and the 2nd place used an optimized U-Net (Isensee et al., 2019). As all the top teams had very similar performances and there were many different detailed strategies in implementation, it is unclear which ones are the dominating factor for the superior performance. One possible strategy is to apply post-processing to our method as the removal of vessels may have a significant impact on the final score. We also participated in the 2017 BraTS challenge using a single model (model 1) and ranked 6th in it [Figure 5 in Myronenko (2019)], showing that the U-Net can be competitive in this challenge with optimization. Further study will be performed on this topic.

For the survival prediction task, since the model is very likely to overfit with the given small dataset and since patient overall survival is affected by many aspects which are not captured in this dataset, we used a multivariate linear regression model as the safest option to minimize overfitting, although at the cost of its expressiveness. As volumetric features are assumed to be most relevant to overall survival, we only included the volumes and surface areas of different sub-regions and ignored other high-order features to reduce overfitting. In addition, these features are easy to interpret as they have direct clinical correspondences; therefore, their clinical adoption can be potentially much easier. This proved to be effective in the challenge; although exploration of additional features and more expressive models with a larger dataset could possibly improve the accuracy of survival prediction. Furthermore, adding other clinical features such as molecular and genetic types may continue to improve the accuracy of prognosis.

In conclusion, we developed an automatic brain tumor segmentation method using an ensemble of 3D U-Nets and showed the superiority over a single model. Based on the segmentation results, we extracted a few simple features and examined their correlations with the overall survival. A multivariate linear regression model was trained to predict the survival and showed high accuracy.

## Data Availability Statement

The datasets generated for this study are available on request to the corresponding author.

## Ethics Statement

Ethical review and approval was not required for the study on human participants in accordance with the local legislation and institutional requirements. The patients/participants provided their written informed consent to participate in this study.

## Author Contributions

XF conducted all experiments and drafted the manuscript. NT, SP, and CM provided significant assistance in technical issues and writing. SP provided guidance in clinical application of this approach and CM provided guidance in the acquisition protocol using MRI.

### Conflict of Interest

The authors declare that the research was conducted in the absence of any commercial or financial relationships that could be construed as a potential conflict of interest.
